# Leveraging Egocentric Social Network Methodology to Understand Risk and Resiliency in Alzheimer’s Disease

**DOI:** 10.1093/geroni/igaf122.904

**Published:** 2025-12-31

**Authors:** Brea Perry, Siyun Peng

**Affiliations:** Indiana University Bloomington, Bloomington, Indiana, United States; Indiana University Bloomington, Bloomington, Indiana, United States

## Abstract

Research suggests social connectedness reduces dementia risk and helps older adults with neuropathology maintain cognitive functionality and quality of life. However, relatively little empirical evidence identifies the specific underlying social and biological mechanisms. This presentation provides an overview of potential pathways through social bridging (i.e., cognitive enrichment through expansive social networks) and social bonding (i.e., neuroendocrine benefits of integration in cohesive social networks). We discuss egocentric social networks, which focus on the structure, function, and composition of ties between a single individual (i.e., the “ego”) and the people they are directly connected to (i.e., “alters”). This methodology is ideal for collecting relational data to operationalize social bridging and bonding theories and test hypotheses regarding risk and resilience in cognitive aging. We illustrate the advantages of egocentric network methods using results from a cohort study of older adults that combines social network data with repeated assessments of general cognitive function and neuroimaging biomarkers. These findings provide insight into specific etiological mechanisms and have important implications for cognitive health disparities that can be leveraged to inform policies and programs that support brain health and cognitive function in older adults.

